# A Preliminary Study on the Relationship between Serum Heparan Sulfate and Cancer-Related Cognitive Impairment: The Moderating Role of Oxidative Stress in Patients with Colorectal Cancer

**DOI:** 10.3390/curroncol29040219

**Published:** 2022-04-12

**Authors:** Danhui Wang, Teng Wang, Min Zhu, Jun Sun, Zhou Zhou, Jinghua Chen, Liping Teng

**Affiliations:** 1 Wuxi School of Medicine, Jiangnan University, Wuxi 214122, China; thomasglan123@163.com (D.W.); 6202807034@stu.jiangnan.edu.cn (M.Z.); woshishuiyayaye@163.com (J.S.); 6212807065@stu.jiangnan.edu.cn (Z.Z.); 2 Department of Oncology, Affiliated Hospital of Jiangnan University, Wuxi 214122, China; drwangteng@163.com; 3 School of Pharmaceutical Sciences, Jiangnan University, Wuxi 214122, China; chenjinghua@jiangnan.edu.cn

**Keywords:** cancer-related cognitive impairment, heparan sulfate, oxidative stress, GSH, colorectal cancer

## Abstract

Cancer-related cognitive impairment (CRCI) has been frequently reported in colorectal cancer survivors. Heparan sulfate (HS) was gradually considered to be related to cognitive disorders. The effect and potential mechanism of HS on CRCI in colorectal cancer patients were unexplored. In this study, all participants were divided into a cognitive impaired group and a cognitive normal group. The concentrations of oxidative stress factors and HS in serum were detected. Associations among HS, oxidative stress factors and CRCI were evaluated. Participants with cognitive impairment exhibited increased levels of HS, GSH, SOD and MDA, compared to the patients with normal cognitive performance. The independent significant association was found between HS and CRCI after controlling for various covariates. The higher concentrations of HS were related to the decreased cognitive performance among survivors who reported higher levels of GSH (β = 0.080, *p* = 0.002). Moreover, the nonlinear association between the level of HS and cognitive scores was confirmed using the restricted cubic splines (*p* < 0.001). These results indicated that the increased concentrations of circulating HS had a nonlinear negative connection with cognitive performance in colorectal cancer survivors, which was moderated by GSH. HS might be a new biomolecule for the identification and management of patients with CRCI.

## 1. Introduction

Non-central nervous system cancer patients often exhibit cognitive symptoms called “cancer-related cognitive impairment (CRCI)”, which is linked to cancer itself and/or its therapy [1]. It is noteworthy that these cognitive symptoms could be one of the most serious negative effects after cancer treatments. CRCI had heavily influenced the negative health-related quality of life and decreased occupational ability and social pressure. Especially, CRCI may persist for even months or years after cancer treatments [2]. Most studies have mainly focused on breast cancer in recent years, resulting in the prevalence of CRCI with a wide range from 16% to 70% in breast cancer patients [3]. Several studies have demonstrated that cognitive impairments were reported by 43% of patients with colorectal cancer [4]. In China, there were a lack of data regarding CRCI in colorectal cancer patients. With the growing demand for long-term cancer care, there is a growing demand for the management of CRCI. Therefore, the underlying mechanisms and therapeutic targets for CRCI should be focused on in colorectal patients for effective early prevention and treatment interventions.

The mechanisms of CRCI are complex and multifactorial. Several hypothesized biochemical pathways have been proposed, but the pathophysiology mechanisms of CRCI are still unclear. Many candidates including oxidative stress, inflammatory response, and blood–brain barrier disruption [5] were put forward, resulting in the alterative profiles of circulating cytokines. Oxidative stress was assessed by measuring malondialdehyde (MDA), nitric oxide (NO), reactive oxygen species (ROS), glutathione (GSH), superoxide dismutase (SOD), glutathione peroxidase (GPx), etc. Furthermore, the previous experimental study showed a dramatic increase in oxidative stress factors such as MDA, or a low level of antioxidants such as GSH in doxorubicin-treated rats with significantly low memory performance [6]. In another study, the ROS was not increased in rat hippocampal neurons treated with cyclophosphamide [7]. These results suggested that the mechanism of CRCI in oxidative stress was still not fully understood. Especially, chemotherapy strongly influenced the cellular process and cell division and interfered with the levels of cytokines. The cytokine investigation in CRCI often implicated that the cytokines were related to other neurocognitive functions [8]. Although researchers have found several cytokines may be induced by chemotherapy and oxidative stress [9], such as higher soluble tumor necrosis factor receptor II (sTNFR II), interleukin-6 (IL-6), tumor necrosis factor-alpha (TNF-α) [10], and others, there is still no effective intervention to improve or predict CRCI.

Heparan sulfate (HS) is a linear, highly charged polysaccharide chain cleaved from heparan sulfate proteoglycans (HSPGs), which is widely presented in macromolecules, normally presented on plasma membranes and in extracellular matrix (ECM) [11]. HS could interact with several partners in ECM or on cell surfaces and connect to nuclear targets to impact the cell cycle and cancer process [12,13]. HS also played an important role in inflammation, immunity, wound healing and morphogenesis due to the multiple interactions [14]. Several studies have shown that HS interacted with tau and β-amyloid aggregates, leading to Alzheimer’s disease (AD) [15,16] or other neurodegeneration and cognitive impairments. Recent studies have shown that HS appears to influence many aspects of the pathogenesis in AD [17,18], and one study has reported that HS might play a role in circadian disruption and phagocytosis of amyloid-beta 42 (Aβ42) [19]. It has been found that septic patients frequently showed cognitive impairment, owing to the interaction between HS fragments in circulation and brain-derived neurotrophic factor (BDNF), leading to inhibiting BDNF-mediated hippocampal long-term potentiation and inducing cognitive dysfunction [20]. In addition, HS and oxidative stress were also closely related. It has been demonstrated that HS was the critical mediator of Aβ-induced oxidative stress and Aβ-induced vascular smooth muscle cell dysfunction [21]. HS accumulation was frequently reported in patients with mucopolysaccharidosis (MPS). It has been suggested that oxidative imbalance might be directly caused by HS accumulation rather than being a secondary consequence of neuroinflammation in patients with MPS [22]. However, the potential role of HS in the development of oxidative-stress-mediated CRCI has been largely overlooked.

Despite the promising evidence that HS may trigger oxidative stress and modulate its relationship with cognitive performance, the moderating role of HS in CRCI has yet to be investigated. Therefore, in the present study, cognitive and neuropsychological assessments, biochemical examinations of serum clinical profiles, and concentrations of serum HS, MDA, NO, GSH and SOD were determined in order to explore whether oxidative stress was involved in the relationship between HS and CRCI among patients with colorectal cancer. The associations among clinical and biochemical data, HS and oxidative stress markers (NO, GSH, SOD, MDA) were analyzed for their influence on CRCI, leading to the better identification and management of CRCI in cancer patients.

## 2. Materials and Methods

### 2.1. Setting and Population

This cross-sectional study was conducted at the Affiliated Hospital of Jiangnan University in Wuxi, Jiangsu, China. The protocol of this study was approved by the Ethical Committee of Affiliated Hospital of Jiangnan University (approval no. LS2020042).

Participants were recruited between June 2020, and June 2021. Eligible candidates were aged over 18 years old, diagnosed with colorectal cancer, and Chinese speaking. Ineligible candidates had a history of stroke, brain metastases, head injury, psychiatric disorders and neurodegenerative disorders. The participants in this study belonged to a subset selected from a large study. In the original study, all participants provided written informed consent. In the progress of the study, patients have the right to reject any part of the study if they have doubts at any time. The initial investigation included 561 cancer patients consisting of 334 colorectal patients. Overall, 334 colorectal patients, who completed the questionnaires, cognitive assessments and provided blood samples, were selected in this study. Finally, a total of 67 patients were randomly enrolled in the subsequent study, which was deemed sufficient to determine statistical power.

### 2.2. Procedure

Functional Assessment of Cancer Therapy—Cognitive Function (FACT-Cog), version 3 and neuropsychological tests were completed on the first day of their regularly scheduled chemotherapy session. Blood specimens for the determination of biomarkers were obtained on the same day. The participants in the current study all provided blood samples and completed the FACT-Cog and neuropsychological tests before chemotherapy administration. Staff members were well-trained in neuropsychological test administration. All blood samples were used to detect the concentrations of serum HS, GSH, SOD, MDA, NO and clinical biochemical data.

### 2.3. Measures

#### 2.3.1. Demographic and Clinical Information

A sociodemographic questionnaire was used to collect information about age, gender, education, body mass index (BMI). Clinical information included tumor stage, blood biochemical parameters such as total cholesterol (TC), triglycerides (TG), albumin, total bilirubin (TBIL), alanine aminotransferase (ALT), aspartate aminotransferase (AST), blood urea nitrogen (BUN), creatinine (Cr), cystatin C (CysC), homocysteine (HCY), carbohydrate antigen 199 (CA199), carbohydrate antigen 125 (CA125) and C-reactive protein (CRP). The results of clinical biochemical blood tests were obtained from the central laboratory of the Affiliated Hospital of Jiangnan University.

#### 2.3.2. Cognitive Performance

Functional Assessment of Cancer Therapy—Cognitive Function (FACT-Cog, version 3 [23,24], Chinese version Cronbach’s α = 0.96 [25]), as a reliable and valid self-report instrument, was used to measure subjective cognitive function, especially for CRCI. This scale included Perceived Cognitive Impairment (PCI; 20 items), Perceived Cognitive Abilities (PCA; 9 items), Comments From Others (OTH, 4 items) and Impact on Quality of Life (QOL, 4 items). A higher score indicated less cognitive impairment. A score decline of 10.6 points compared with normative data from the healthy populations was considered clinically meaningful [26].

#### 2.3.3. Neuropsychological Assessment

The neuropsychological assessment included 3 fields of cognitive function, attention function: the Digit Span subtest of Wechsler Adult Intelligence Scale-III (WAIS-III), processing speed and executive function: Trail Making Test (TMT A), memory and learning function: Hopkins Verbal Learning Test-Revised (HVLT-R). The validity and reliability of the Chinese version of the test in this study have been well determined. The raw scores of these three tests were converted to Z scores based on the age-matched normative data in a healthy Chinese population [27]. The neuropsychology test score ≤ 1.5 standard deviations was defined as “cognitive impairment”.

WAIS-III in this study was used to administer a forward task to measure the attention function. The score was calculated when patients correctly repeated sequences of the whole number after two failed attempts each time [28].

TMT A was performed to measure a patient’s visual scanning and searching abilities, processing speed, mental flexibility and executive function. Patients were asked to draw connecting lines among encircled numbers in sequentially on the paper [29].

HVLT-R was used to assess learning and memory function. The individual was asked to listen and repeat the word and list as many words as possible. This progress was conducted 3 times, followed by free recall. Correct responses through these three learning and memory tests were summed for the whole scores (range 0–36) [30].

#### 2.3.4. Detection of Serum Biomarkers

Blood specimen was collected before breakfast by the licensed phlebotomist. The blood samples were centrifuged for 15 min at 2500 rpm to obtain serum. At least 600 μL serum of each specimen was stored at −80 °C. The concentrations of NO, MDA, GSH and SOD in serum samples were determined using the commercial kits (Beyotime, Shanghai, China), according to the manufacturer’s instructions.

The concentration of HS in serum was measured following the published method with some modifications [31]. Briefly, purified HS standard substance with different concentrations was used to form a standard curve, and HS concentration was identified from the standard curve equation. Specifically, a 250 μL serum sample with 2.5 mL of 1,9-dimethylmethylene blue (DMMB, Sigma, St. Louis, MO, USA) reagent and 250 μL deionized water were added to directly measure the absorbance at 525 nm using a microplate reader. The HS concentration was expressed as micrograms per milliliter of serum.

### 2.4. Statistical Analyses

The entire cohort of 67 participants was calculated for statistics and data analysis. Normally distributed data (continuous variables) were expressed as means and standard deviations and analyzed using a two-tailed, two-sample t-test and one-way analysis of variance (ANOVA) for comparisons in two groups. Skewed distribution data were described as median (interquartile range) and analyzed using the Mann–Whitney U test for comparisons. Benjamini–Hochberg (BH) [32] was used to adjust *p* values, and *P*_BH_ < 0.05 was considered statistical significance. Categorical variables were reported as frequency and percentage and analyzed using the Chi-square test. Spearman correlation coefficient was calculated to determine the relationship between variables. Statistics significance was considered as *p* < 0.05.

To examine the independent association between serum HS concentration and FACT-Cog scores, the multivariate linear regression was conducted to adjust covariates in three models. In the crude model, no variable was adjusted. In model 1, the well-known potential factors (age, gender, and education) of FACT-Cog scores were adjusted. In model 2, serum GSH and SOD were enrolled as the adjustment factors because there were significant differences between the two groups. The potential confounders were added in the adjustment: age, gender, education, GSH and SOD.

To determine whether oxidative stress markers as a moderator variable (W) moderated the relationship between FACT-Cog scores (Y) and HS concentrations (X), moderation analysis was performed after adjusting the covariates (age, education and gender). Tests of simple slopes were performed to compare three levels of GSH concentration including high (+1 SD), mean (m), low (−1 SD). The moderation model was built using the R package (gvlma and sjPlot). Furthermore, the hypothetical non-linear relationship between HS and cognitive performance was evaluated by restricted cubic splines. In this nonlinear model, HS concentration was allowed to be adjusted for GSH, age, gender and education.

All statistical analyses were conducted in R 4.0.2. Inferential statistical analyses were statistically significant at an alpha level of 0.05. The power calculation was used G power software. The value of α = 0.05, the effect size (r) was 0.34, and the power of this study was 83%. The interpretation of medium effect size (r) was >0.3, and large effect size was >0.5 [33].

## 3. Results

### 3.1. Social Demographic Profile, Cognitive Performance, Clinical and Blood Biochemical Characteristics of Study Participants

In this study, a total of 67 participants (60% male and 40% female) provided informed consent and cognitive assessment data. Participants with FACT-Cog scores less than 101 points were selected into the “cognitive impaired group”; otherwise, they were assigned to the “cognitive normal group”. The demographic and clinical data of the cognitive impaired group and the cognitive normal group are shown in Table 1. Table 1 also lists the *p* values before and after adjusting BH. The median age of the participants was 60 years old (the interquartile range (IQR), 54.0 to 66.5). Based on the FACT-Cog scores, participants were divided into two groups: 32 survivors (47.76%) with cognitive impairment and 35 normal cognitive survivors (52.23%). Participants in the cognitive impaired group were more likely to have declined attention function [−1.16 (−1.59, −0.63)], processing speed and executive function [−0.4 (−1.19, 0.88)] and learning and memory function (0.52 ± 1.31) compared to the cognitive normal group. Although there were no significant differences in neuropsychological tests between the two groups, the patients in the cognitive impaired group had a worse cognitive performance. In addition, demographics and neurocognitive tests had no differences in the two groups after adjusting BH. As shown in Table 2, these two groups had no significant differences in general variables (age, gender, education, BMI) or some clinical biochemical data (TG, TBIL, ALT, AST, Albumin, BUN, Cr, HCY, CA199). However, the cognitive impaired group had higher TC (*p* = 0.001, *P*_BH_ < 0.01), CysC (*p* = 0.03, *P*_BH_= 0.101), CRP (*p* < 0.001, *P*_BH_ < 0.001) and CA125 (*p* = 0.008, *P*_BH_ = 0.036) concentration than the cognitive normal group in colorectal cancer survivors, but the *P*_BH_ value of CysC showed no significant differences in two groups.

### 3.2. Serum Levels of HS and Oxidative Stress Factors in Participants

**Figure 1:**
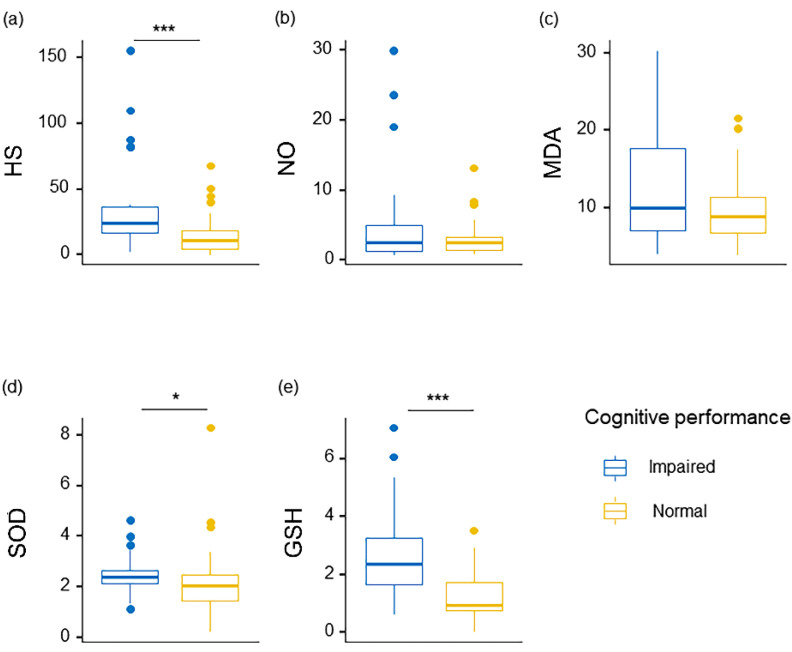
The levels of (**a**) HS, (**b**) NO, (**c**) MDA, (**d**) SOD and (**e**) GSH in the serum of “Impaired” (blue circles) and “Normal” (yellow circles) cognitive performers. Colorectal cancer individuals with distinct cognitive performances presented differences in the concentrations of HS and oxidative stress factors. Dots represent each participant, columns represent the mean of the group, and bars represent the standard error of the mean (* *p* < 0.05, *** *p* < 0.001).

Serum HS concentration and biomarkers of oxidative stress (NO, MDA, SOD, GSH) in the two groups are shown in Table 3, and the comparisons of these factors are visualized in Figure 1. The cognitive impaired group had higher (*p* < 0.05) concentrations of serum HS, SOD and GSH than the cognitive normal group. Especially, the cognitive impaired group had significant increases in circulating levels of HS and GSH (*p* < 0.001, *P*_BH_ < 0.001). After adjustment for BH, serum SOD in these two groups showed no significant differences. Although the pairwise comparisons of serum MDA (*p* = 0.23) in these two groups had no significant differences, median values of the measure showed a higher-expression trend in the cognitive impaired group than those in the cognitive normal group. Similar to the neurocognitive scores, the cognitive impaired group presented a growing and progressive tendency in oxidative stress biomarkers.

### 3.3. Associations among Serum HS, Oxidative Stress Factors and Cognitive Impairment

**Figure 2:**
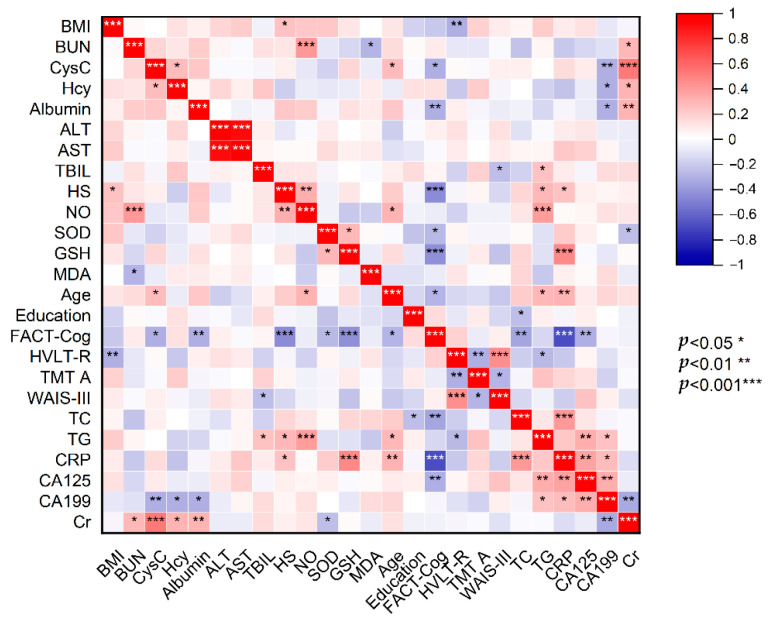
Heat map displaying correlations of cognitive scores with HS, clinical biochemical data and oxidative stress biomarkers. The scale with the bipolar color progression from red to blue on the right of the figure is used to indicate different correlations from positive to negative correlations. Asterisks indicate statistical significance (* *p* < 0.05, ** *p* < 0.01, *** *p* < 0.001).

The relationship of cognitive scores, serum HS, serum biomarkers of oxidative stress and variety of clinical laboratory blood parameters is displayed in the heat map (Figure 2). The concentration of serum HS was negatively correlated with cognitive scores (r = −0.34, *p* < 0.001) but positively correlated with the value of serum NO (r = 0.33, *p* < 0.01), TG (r = 0.28, *p* < 0.05) and CRP (r = 0.24, *p* < 0.05). Furthermore, the cognitive scores seemed to have a significantly strong correlation with HS, SOD, GSH, CysC, albumin, CRP, TC, CA125 and age, and all these correlations (r = −0.26 to −0.68, *p* < 0.05 to 0.001) were negative. Reciprocally, the value of serum GSH was positively correlated with circulating levels of SOD (r = 0.3, *p* < 0.05) and CRP (r = 0.47, *p* < 0.001), and negatively correlated with cognitive scores (r = −0.44, *p* < 0.001). The concentration of SOD was also negatively correlated with the value of Cr (r = −0.24, *p* < 0.05) and cognitive scores (r = −0.26, *p* < 0.05). Likewise, the value of NO was positively correlated with the concentration of BUN, age, TG and HS (r = 0.3 to 0.41, *p* < 0.05 to 0.001). The value of MDA was only negatively correlated with BUN (r = −0.29, *p* < 0.05).

In multivariable regression models, the association between cognitive scores and HS was further verified (Table 4). In the crude model, a higher level of HS was significantly associated with severe cognitive impaired function (point estimate = −0.28, 95% confidence interval = −0.46 to −0.09, *p* = 0.003). In Model 1, with adjustment for sex, gender and education, the significant association remained (point estimate = −0.26, 95% confidence interval = −0.45 to −0.08, *p* = 0.005). Additionally, in model 2, after further adjustment for GSH and SOD, HS still had a strong correlation with cognitive scores (point estimate = −0.23, 95% confidence interval = −0.41 to −0.06, *p* = 0.01). Interestingly, GSH was associated with cognitive scores (point estimate = −4.329, 95% confidence interval = −7.81 to −0.84, *p* = 0.01) after controlling for gender, age, education and SOD.

### 3.4. Association between Cognitive Impaired Performance and HS as Moderated by Oxidative Stress Markers

**Figure 3:**
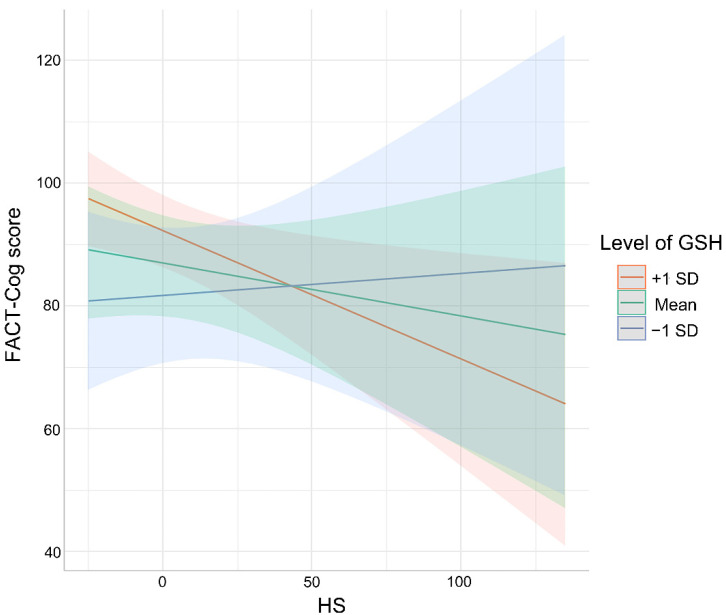
Predicted probability of GSH as a moderator of the relationship between HS and cognitive scores. Elevated HS was associated with poor cognitive performances only among patients who reported a high concentration of GSH. Models were adjusted for age, gender and education. Shaded areas depict confidence intervals of simple slopes.

**Figure 4:**
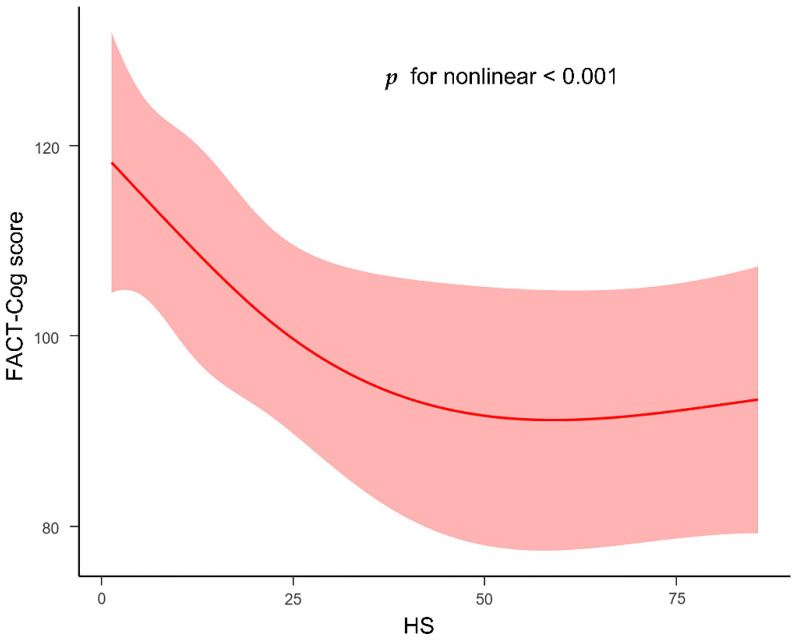
The nonlinear association between HS and cognitive scores. Analyses were adjusted for GSH, age, gender and education. Linear regression models with restricted cubic splines with a 95% confidence interval (CI) were used.

Furthermore, the hypothesis that oxidative stress markers would moderate the association between HS and cognitive scores was confirmed by setting moderation models. As shown in Figure 3, indeed, only GSH was found to moderate the association between HS and cognitive scores (SE = 0.05, unstandardized regression coefficient β = 0.12, *p* = 0.02), such that HS was negatively associated with cognitive scores among participants who reported high (+1 SD) levels of GSH (SE = 2.38, unstandardized regression coefficient β = −8.79, *p* < 0.001) and middle (mean) levels of GSH (SE = 1.68, unstandardized regression coefficient β = −5.28, *p* < 0.01). In contrast, the association between HS and cognitive impaired performance was non-significant for patients with low (−1 SD) concentrations of serum GSH (SE = 2.1, unstandardized regression coefficient β = −1.76, *p* > 0.05). Nevertheless, the values of SOD, MDA and NO were all enrolled in the moderated tests, showing the non-moderated effects on the association between HS and cognitive impairment. To further quantify the dose–response relationship between HS and cognitive performance, the restricted cubic spline was conducted after being adjusted for GSH, age, gender and education. The distinct nonlinear relationship between HS and cognitive scores (*p* < 0.001) is presented in Figure 4.

## 4. Discussion

The prevalence of cognitive decline has been reported to reach an average of 32% in colorectal cancer survivors [34]. More attention should be paid to colorectal cancer patients with cognitive impairment. However, it was reported that lower scores in neuropsychology tests often showed no association with cognitive complaints [35]. In line with the previous study, the current study showed a higher prevalence of colorectal cancer survivors with cognitive impairment (47.76%), which had no significant correlation with the objective results of neuropsychology tests. Cognitive impairment might affect daily life or affect/induce other symptoms (such as anxiety and depression) [35], suggesting that patients with cognitive decline complaints should be highly concerned. It was found that the risk factors of the increased prevalence of CRCI were normally involved in social–demographic factors, cancer-therapy-related factors, psychological factors, and some cytokines including IL-6, TNF-α, sTNRFII, etc. [36]. Although the effect of age on CRCI was proven [37], it was difficult to distinguish whether cognitive symptoms were age-related or cancer-induced. In addition, social-demographic risk factors of CRCI also included education level [38]. However, in the present study, the cognitive impaired group and the cognitive normal group both have been found that there were no differences in age and education level. Furthermore, the biomarkers including cytokines and inflammation factors have been involved in cognitive decline in cancer patients [8]. In the present study, the relationship between clinical circulating biomarkers and cognitive decline in cancer patients was explored. Surprisingly, CRP, CysC, TC and CA125 were found to be dramatically higher in the cognitive impaired group compared with those in the cognitive normal group in colorectal cancer patients. Consistent with previous studies, cancer survivors with cognitive impairment had a higher level of inflammation biomarkers [36]. A recent study supported the evidence that chemotherapy released the inflammation cytokines to damage the brain structure and cause the activation of microglia [39]. There were several studies that focused on the dramatical association between CysC and cognitive impairment [40,41], resulting in the evidence that impaired renal function would influence cognitive decline. Likely, the present study showed similar results in colorectal cancer patients, providing the possibility that renal function might be associated with cognitive decline in colorectal cancer.

Several possible mechanisms of CRCI have been found, including oxidative stress, blood–brain barrier compromise and inflammation. It has been reported that the insufficiency of antioxidant mechanisms would make the central nervous system susceptible to ROS attack. Especially, chemotherapy-induced intensifying oxidative stress and reduced antioxidative enzymes, resulting in cognitive impairment [42]. Similarly, Chenying Wang and colleagues found DOX treatment-induced cognitive impairment in rats with cancer, indicating that the level of MDA was obviously elevated and the reduction in GSH and SOD activity existed [43]. In contrast, there were also several studies that reported the opposite results. For instance, Carolina Vieira Cardoso et al. reported an increasing level of NO, GSH and lipid oxidation in DOX-treated rats with cognitive impairment [44]. Ciara Bagnall-Moreau and colleagues found that, after AC (doxorubicin and cyclophosphamide) chemotherapy, the higher expression of several oxidative stress and antioxidative genes was presented in rats with chemotherapy-induced cognitive impairment [7]. In the present study, oxidative stress markers including MDA, SOD and GSH were evaluated. In the cognitive impaired group, all these markers were higher than those in the cognitive normal group. The higher level of oxidative factors (MDA) in the cognitive impaired group was in line with the findings from the previous studies. The present study also showed that cognitive impairment was related to the rising level of antioxidative markers (GSH and SOD). It was consistent with the previous studies that a dramatical elevation of antioxidants was related to cognitive decline in older adults [45] and patients with schizophrenia [46]. The possible hypothesis to explain this phenomenon was that the increase in antioxidative response might reflect the compensatory effects of the body. It has been indicated by several animal studies that overexpression of SOD in murine prevented the role of caspase-activated mitochondrial apoptosis to protect susceptible neurons [47].

Several studies have examined the relationship between HS and cognitive impairment in other diseases. One early study has shown that HS was related to AD [48]. In recent years, it was shown that the over-expression of HS caused cognitive dysfunction in AD [49]. Kaori et al. [20] found that circulating HS fragments (2-O- and N-sulfated HS) led to cognitive impairment in septic patients. Another review reported that excessive HS levels affected the process of neurotransmission even more CNS function in patients with mucopolysaccharidosis [50]. It is noteworthy that recent studies have suggested that some neurocognitive symptoms were significantly improved by reducing the level of HS [51,52]. In the present study, the level of HS had a significant independent association with CRCI. Furthermore, the cognitive impaired group had higher circulating HS compared to the colorectal cancer patients with normal cognitive function. It has been revealed that HS participated in the progress of interactive function with Aβ and led to oxidative stress response [21]. It also has been proposed that the accumulation of HS could induce the secondary retention of oxidative products, especially in the brain [22]. Likewise, the present study provided the moderating function among HS, CRCI and oxidative stress. Elevated HS was associated with worse cognitive performance only among participants who had a higher level of GSH. After adjusting for the concentration of GSH, it was found that there was a nonlinear relationship between HS and cognitive function. Taken together, these findings indicated that circulating HS might be significantly related to the cognitive impairment in colorectal cancer patients, which might provide a new marker for the diagnosis and the potential target to remit CRCI.

Strengths of the current research presented the relationship between cognitive performance and serum markers including serum HS, oxidative stress markers and clinical biochemical parameters in colorectal cancer patients. The level of serum HS had a dramatic relation with the cognitive scores. The relationship between circulating HS and cognitive scores was moderated by the oxidative stress marker GSH. Additionally, the concentration of serum HS had a nonlinear relationship with cognitive scores, suggesting that, with a higher level of HS, the worse cognitive performance was presented, and that the highest concentration of HS would not influence the worst cognitive symptoms. Furthermore, the present study included patients from the clinical environment and combined the clinical data to deeply explore the mechanism of CRCI. Circulating HS, which is prevalent in other cognitive disorder diseases, might be a potential marker in the management of CRCI patients.

Nevertheless, the present study should be considered in several limitations. First, this was a cross-sectional study, and prospective screening or animal experiments should be conducted in the future to further verify the findings from this present study. Second, it was probably to find a significant relationship between HS and CRCI, but current theories did not provide an explanation for how HS would interact with cognitive impairment in cancer patients. Further work should gain deeper insight into the mechanism of HS, GSH and CRCI. Third, in this study, the assessment did not control for other correlated factors such as fatigue, sleep, pain, depression and anxiety, which can also influence CRCI. In future studies, these symptoms, especially psychoneurological symptom clusters, should be identified in colorectal cancer patients with CRCI. Fourth, the results were focused on adults with colorectal cancer, and it should be evaluated whether there were the same findings in other cancers. Moreover, the relationship between HS and cognitive impairment in other cancer populations needs to be explored.

## 5. Conclusions

The results from the present study showed a significant independent association between circulating HS and CRCI, as moderated by GSH. The rising level of HS was associated with reduced cognitive status in colorectal cancer patients with a higher level of GSH. These findings shed light on a new potential marker (HS) for CRCI, provide the risk factors of CRCI and reveal the potential mechanism of oxidative stress markers in the relationship between HS and CRCI. Future studies need to investigate how HS interacts with GSH, providing clues on potential strategies to better identify and manage CRCI.

## Figures and Tables

**Table 1:** Demographics and neurocognitive tests of the participants.

Variables	Total (*n* = 67)	Cognitive Impaired Group (*n* = 32)	Cognitive Normal Group (*n* = 35)	*p*	Adjusted *p*
Age, years	60 (54, 66.5)	62.5 (57, 69)	57 (53, 66)	0.08	0.252
BMI, kg/m^2^	22.06 (20.36, 25.08)	22.65 (20.97, 25.11)	21.85 (20.34, 24.69)	0.38	0.544
Gender, *n* (%)				0.76	0.824
Male	40 (60)	18 (56)	22 (63)		
Female	27 (40)	14 (44)	13 (37)		
Education, *n* (%) years				0.48	0.615
6	17 (25)	10 (31)	7 (20)		
9	22 (33)	11 (34)	11 (31)		
12	17 (25)	5 (16)	12 (34)		
15	6 (9)	3 (9)	3 (9)		
16	5 (7)	3 (9)	2 (6)		
Cancer staging, *n* (%)				0.23	0.482
1	2 (3)	1 (3)	1 (3)		
2	9 (13)	7 (22)	2 (6)		
3	32(47.8)	13 (41)	19(54)		
4	24 (36)	11 (34)	13 (37)		
HVLT-R	0.69 ± 1.18	0.52 ± 1.31	0.86 ± 1.04	0.25	0.482
TMT A	−0.62 (−1.17, 0.12)	−0.4 (−1.19, 0.88)	−0.64 (−1.14, −0.01)	0.37	0.544
WAIS-III	−1.13 (−1.59, −0.12)	−1.16 (−1.59, −0.63)	−0.48 (−1.59, −0.02)	0.38	0.544
FACT-Cog	102 (77, 115.5)	74 (69, 90.25)	114 (104, 127.5)	<0.001	<0.001

**Table 2:** Blood biochemical parameters of the participants.

Variables	Total (*n* = 67)	Cognitive Impaired Group (*n* = 32)	Cognitive Normal Group (*n* = 35)	*p*	Adjusted *p*
TC, mmol/L	4.24 ± 0.65	4.50 ± 0.64	4.00 ± 0.57	<0.01	<0.01
TG, mmol/L	1.76 (1.39, 2.51)	1.98 (1.7, 2.26)	1.65 (1.18, 8.61)	0.47	0.616
CRP, mg/L	1.70 (1.45, 2.83)	2.85 (2.61, 3.28)	1.48 (1.38, 1.53)	<0.01	<0.001
CA125, U/mL	13.16 (7.64, 28.3)	17.48 (9.18, 56.61)	10.67 (6.85, 15.25)	<0.01	0.036
CA199, U/mL	11.82 (8.11, 24.45)	12.07 (7.84, 83.09)	11.77 (8.34, 18.7)	0.593	0.696
TBIL, μmol/L	9.30 (7.65, 13.17)	10.25 (7.65, 13.47)	8.99 (7.85, 12)	0.7	0.789
ALT, U/L	18.39 (15.5, 29.5)	19.27 (12.5, 30)	18.35 (16.12, 25)	0.92	0.482
AST, U/L	21.00 (18.55, 29)	24.00 (18.75, 34)	20.00 (18.55, 23.56)	0.2	0.961
Albumin, g/L	41.67 ± 4.02	42.20 ± 4.77	41.18 ± 3.18	0.31	0.544
BUN, mmol/L	4.88 (4.31, 5.67)	4.69 (3.78, 5.48)	5.00 (4.52, 5.87)	0.23	0.482
Cr, μmol/L	69.76 ± 15.25	69.70 ± 13.66	69.82 ± 16.77	0.97	0.97
CysC, mg/L	0.98 ± 0.22	1.04 ± 0.22	0.92 ± 0.21	0.03	0.101
HCY, μmol/L	12.16 (11.82, 12.72)	12.01 (11.5, 12.81)	12.23 (11.94, 12.64)	0.33	0.544

**Table 3:** Comparisons of serum HS and related oxidative stress markers between the two groups of participants.

Variables	Total (*n* = 67)	Cognitive Impaired Group (*n* = 32)	Cognitive Normal Group (*n* = 35)	*p*	Adjusted *p*
HS, μg/mL	17.31 (5.39, 31.43)	24.50 (16.48, 36.37)	10.93 (4.26, 18.17)	<0.001	<0.001
GSH, μmol/L	1.63 (0.96, 2.85)	2.35 (1.67, 3.25)	0.96 (0.76, 1.74)	<0.001	<0.001
SOD, U/mL	2.32 (1.71, 2.61)	2.37 (2.12, 2.66)	2.07 (1.43, 2.46)	0.026	0.100
MDA, μmol/L	9.42 (6.92, 12.73)	10.01 (7.06, 17.63)	8.84 (6.81, 11.35)	0.231	0.482
NO, μmol/L	2.54 (1.33, 4.25)	2.46 (1.27, 4.93)	2.56 (1.43, 3.21)	0.589	0.696

**Table 4:** Adjusted associations between HS and cancer-related cognitive impaired performance tested by FACT-Cog scores.

	Point Estimate	CI	Std. Error	*p*
Crude	−0.28298	−0.4666478 −0.09931496	0.09196	0.00306
Model 1	−0.26614	−0.4506025 −0.08168605	0.09228	0.00539
Model 2	−0.23739	−0.4186311 −0.05614362	0.09061	0.0111

Model 1: Crude Model + adjustments for age, gender and education. Model 2: Model 1 + adjustments for GSH and SOD.

## Data Availability

The data presented in this study are available on request from the corresponding author. The data are not publicly available due to privacy.
